# Superficial Peritoneal Endometriosis Vaporization Using a CO_2_ Laser: A Long-Term Single-Center Experience

**DOI:** 10.3390/jcm13061722

**Published:** 2024-03-17

**Authors:** Stefano Di Michele, Silvia Bramante, Stefano Angioni, Michela Bernassola, Tommaso De Vita, Daniela Anna Iaccarino, Luca Giannoni, Maurizio Rosati

**Affiliations:** 1 Division of Gynecology and Obstetrics, Department of Surgical Sciences, University of Cagliari, 09124 Cagliari, Italy; dr.dimichelestefano@gmail.com (S.D.M.);; 2 Unit of Obstetrics and Gynecology, Santo Spirito Hospital, 65129 Pescara, Italy; silviabramante@hotmail.com; 3 Department of Obstetrics and Gynecology, SS. Annunziata Hospital, G. D’Annunzio University of Chieti-Pescara, 66100 Chieti, Italy; 4 El.En. Group, 50041 Calenzano, Italy; l.giannoni@elen.it

**Keywords:** endometriosis, superficial peritoneal endometriosis, CO_2_ laser, pain, fertility

## Abstract

**Background**: The validation of laser usage during laparoscopic procedures, notably by Camran Nezhat in the late 1980s, has been significant. Lasers offer precision and depth control in tissue vaporization without bleeding. Surgical intervention remains central in managing endometriosis-associated pain and infertility, especially for patients unresponsive to hormonal therapy. **Methods**: This retrospective cohort study included 200 patients with superficial peritoneal endometriosis (SPE) who underwent laparoscopic laser vaporization. Surgery was performed using a CO_2_ laser, and histological confirmation of endometriosis was obtained for all cases. Pain scores and SF-36 questionnaire domains were assessed preoperatively and postoperatively. Fertility outcomes were evaluated among patients desiring pregnancy. **Results**: Significant improvements in pain score and SF-36 questionnaire domains were observed postoperatively (*p*-value < 0.01), indicating enhanced quality of life. Among infertile patients with an active desire for pregnancy, surgical treatment showed an overall pregnancy rate after surgery of 93.7% (*p*-value < 0.01), including 75.7% natural pregnancies and 24.3% IVF. Laser vaporization enabled precise lesion removal with minimal tissue damage, short operative time, and minimal blood loss. **Conclusions**: Laparoscopic laser vaporization is an effective treatment for SPE, offering pain relief, improved quality of life, and favorable fertility outcomes. Further research is needed to validate these results in terms of pain control and fertility.

## 1. Introduction

Current advances in genetic pattern studies have enhanced our understanding of endometriosis variability, pain perception, persistence, and related inflammatory conditions. Targeted exploration of genetically regulated mechanisms shared between endometriosis and other pain disorders is essential for the development of novel treatments and the facilitation of early symptomatic intervention [1]. Endometriosis is a benign, chronic estrogen-dependent, neuroinflammatory condition characterized by the growth of endometrial-like tissue (‘lesions’) outside of the uterus, most commonly on the peritoneum and ovaries [2]. Approximately 10 percent of all reproductive-aged worldwide women are affected by endometriosis [3]. Adolescents are not exempt from endometriosis, and suspicion of the condition in this age group warrants prompt medical evaluation to initiate treatment, both medical and potentially surgical, before the condition progresses to a more severe stage [4]. Although several theories have been proposed, the pathogenesis of endometriosis is still under debate, and a definitive cause remains unknown [5]. The most favored one describes how retrograde menstruation through the fallopian tubes could invade the peritoneal mesothelium, leading to diffuse implants of the disease and its blood supply for survival and growth [6,7]. The stem cell theory for endometriosis pathogenesis, supported by the consensual mechanism of retrograde menstruation, highlights the recognized importance that menstrual blood-derived stem cells have gained by potentially being directly related to the genesis, development, and maintenance of ectopic endometriotic lesions [8]. Endometriosis can be disabling and strongly compromise life quality along with the critical associated social-economic cost. The identification of heightened nerve fiber presence in areas with increased macrophage counts suggests a correlation between macrophage density and the quantity of nerve fibers. This correlation, in turn, appears to be linked to the manifestation of symptoms associated with endometriosis [9,10]. Moreover, it is noted that the release of nerve growth factors leads to changes in the peritoneal innervation [9]. Three endometriosis subtypes have been described through the years: SPE, ovarian endometriosis, and deep endometriosis [11]. Pain-related symptoms and infertility are the most common indications for surgery among affected patients. Before surgery, hormonal therapy should be attempted as a therapeutic measure, particularly in cases lacking sonographic evidence of endometriosis, to elucidate the source of pain and to identify patients with symptomatic peritoneal endometriosis. Surgical procedures should be undertaken to alleviate pain and enhance pregnancy rates. In the case of infertility, there is a lack of evidence about whether surgical management should be used as the first intervention or only in cases where medically assisted reproduction treatment (ART) has failed [12,13]. Pain intensity is not influenced by the stage or extent of the disease, or the appearance and location of endometriosis deposits [14,15]. Approximately 80% of individuals with endometriosis experience superficial peritoneal localization. Eventually, a definitive diagnosis, due to the absence of non-invasive tests for endometriosis, is achieved through the visualization of lesions during diagnostic laparoscopy [16]. In cases where superficial peritoneum endometriosis is identified during laparoscopy, gynecologists often decide on surgical removal through excision or ablation [17,18]. The investigation and surgical removal processes demand specialized gynecological skills; nevertheless, about 50% of patients who undergo surgical treatment for endometriosis encounter persistent or recurrent pain within five years, leading to high rates of surgical reintervention [13,19]. Moreover, the recurrence rate of clinically detectable endometriosis tends to be higher in older women with advanced stages of the disease and lower in women with infertility [20]. From the very beginning, the use of CO_2_ laser laparoscopy became popular and effective in the treatment of endometriosis [21,22,23]; thanks to its versatility, many different techniques, such as hydro-dissection, were developed, leading to a safe laser endoscopic excision or vaporization, of peritoneal endometriosis [22]. There is insufficient evidence to substantiate the effectiveness of current endometriosis guidelines in determining whether surgical removal of isolated SPE enhances or deteriorates symptoms and quality of life [17,18]. Laser vaporization of endometriotic lesions remains an excellent tool for laparoscopic surgeons; a recent review and meta-analysis showed a comparison among different surgical techniques in women with ovarian endometrioma, resulting in no differences in recurrence rate and pregnancy rates. Still, the antral follicle count was higher in the laser vaporization group [24]. There is an ongoing multicenter trial in the UK known as ESPriT2. In this trial, women diagnosed solely with SPE during diagnostic laparoscopy are randomly assigned to either undergo surgical removal of SPE or receive diagnostic laparoscopy alone. The objective is to ascertain whether the surgical removal of endometriotic lesions enhances overall symptoms associated with endometriosis and improves quality of life or if surgery offers no discernible benefits, worsens symptoms, or potentially causes harm [25]. This paper focuses on our experience and presents the follow-up (FU) results in terms of pain, fertility outcome, and recurrence rate of women affected by SPE after laparoscopic laser vaporization.

## 2. Materials and Methods

### 2.1. Study Design and Population

We analyzed our single-center database to identify all women who underwent laparoscopic procedures between January 2014 and December 2020 at our endometriosis center at the gynecology department, S. Spirito Hospital of Pescara, Italy. This retrospective cohort study was conducted with the approval of the Ethics Committee of our institution and was performed in line with the principles of the Declaration of Helsinki. Informed consent was obtained from all subjects involved in the study. Endometriosis was diagnosed based on symptoms and clinical examination and confirmed by transvaginal sonography (TVS) and/or pelvic magnetic resonance imaging (MRI) when needed. Patients were not excluded if they had already been diagnosed with endometriosis. Symptomatic patients with or without impaired fertility older than 18 years, with a sonographic exclusion of complex endometriosis, were included in our study. Any patient with previous ovarian cysts or endometriomas or any signs of deep infiltrating endometriosis (DIE) or adenomyosis was excluded. Our patient selection aimed to focus the attention on women with only superficial peritoneal diffusion of endometriosis. Surgical indications included symptomatic lesions with a suboptimal response or intolerance to medical treatment (progesterone or combined hormonal contraceptives), particularly the presentation of pain symptoms with or without infertility. Only patients with histologically confirmed endometriosis were included in our analysis. 

### 2.2. Surgical Procedure

All surgical procedures were performed by senior surgeons experienced in minimally invasive surgery for endometriosis (M.R., S.B.) using a scanning-aided CO_2_ laser: Smartxide2 C80H (DEKA m.e.l.a. Calenzano, Italy). The laser has a radiofrequency (RF) excited ultra-pulsed laser source, producing up to 80 W of maximum average power. Scanning technology moves extremely fast (up to 1 millionth of a second of dwell time). The laser beam is focused on the target using a fixed focal coupler, passing through the operative channel of the Laparoscope (we used the STORZ 26075AA model). This allows the delivery of energy on tissue in a controlled and repeatable manner. Scanning-aided “free beam” laser surgery is the only technology that enables the surgeon to control the ablation depth and the thermal effect for each scanning passage. Moreover, scanning technology has already been intensively used in many fields of surgery like Gynecology, Colposcopy, Ears-Nose-Throat, Neurosurgery, and General Surgery (i.e., Proctology and Wound Healing) [26,27,28,29,30]. The CO_2_ laser can also be delivered using a flexible hollow fiber (i.e., Smartxide2 TRIO System, DEKA, Calenzano, Italy). Nevertheless, the fiber, even if very ergonomic and helpful for precision cutting in near contact mode, does not have the control guaranteed by scanning-aided technology for vaporization. To vaporize tissue with the fiber, the surgeon must significantly move the tip back from the target to defocus the laser beam. Then, the defocused spot is moved manually, thus producing an unpredictable depth of ablation and higher and uncontrolled thermal damage. In all cases, a careful evaluation of the whole abdominal cavity and a dye test to assess tubal patency was performed. The clinically suspected diagnosis was verified during surgery, and all visible endometriosis implants, along with inflammatory altered peritoneum, were treated. We systematically conducted a SPE biopsy by creating a circular incision with a 1 to 2 cm margin around the lesion. The peritoneum was then grasped using an atraumatic forceps and peeled away with the assistance of a laser. This approach aimed to confirm the diagnosis of endometriosis and allow for analysis of inflammation and fibrosis status by pathologists reviewing the submitted histological sample. SPE treatment was carried out using direct and accurate CO_2_ laser vaporization with minimal tissue damage without injury to adjacent structures. In cases where anatomy was distorted by adhesions and the surgical approach became more challenging, as a precautionary measure, we performed the laser vaporization of endometriotic lesions using the hydro-dissection technique [22]. In cases where patients’ clinical conditions permitted, we performed laparoscopic laser vaporization under conscious sedation, a standardized approach in our center, due to the longstanding collaboration between the surgical and anesthesiologic teams [31,32]. 

### 2.3. Data Collection

We reviewed the patients’ records in our study to collect data about preoperative, intraoperative, postoperative, and FU evaluation reports. The routine presurgical assessment consisted of the collection of medical history data, physical and vaginal pelvic examination, TVS, and/or MRI. The patient’s age, body mass index (BMI), previous abdominal surgery, indication for surgery, and previous medical treatment were assessed among the preoperative data. Intraoperative parameters were collected, including overall operating time, blood loss, conversion rate, and complications. Operative time was conventionally defined as the time from skin incision to skin closure. The estimated blood loss (EBL) was calculated by the difference in the total quantities of suctioned and irrigation fluids at the end of the surgical procedure. Intraoperative complications were recorded based on a classification of intraoperative complications [33]. Postoperative parameters that were collected included postoperative pain, time to discharge, early complications (within 30 days of the procedure), and late complications (>30 days), measured according to the Clavien–Dindo Classification of Surgical Complications Scale [34]. The preoperative pelvic pain severity was assessed using a 10-point visual analog scale (VAS) that was routinely performed at preoperative visits and covered different types of pain: dysmenorrhea, chronic pelvic pain, dyspareunia, dysuria, and dyschezia. Vas scores have been validated as a reliable method for pain assessment and were employed to gauge both overall pelvic pain and various types of visceral pain, stating all clinically relevant symptoms with a score of ≥5. Moreover, for the patients attending our center, their quality of life and health-related gratification were routinely measured with the Medical Outcomes Survey Short Form 36 (SF-36), the most fully applied common instrument for evaluating health-related quality of life [35]. We used the revised score of the American Society of Reproductive Medicine [36] (rASRM) to classify different stages of the disease. 

### 2.4. Follow-Up 

Systematic postoperative, clinical, and symptomatic assessments were achieved in six months, one year, and two years. At each FU visit, a complete evaluation consisted of a patient interview to define subjective symptoms, administration of a validated questionnaire, a gynecological pelvic investigation, and a TVS evaluation. The primary outcome was the confirmation of diagnosis, a change in pain symptoms, and quality of life assessment. The secondary outcome measures were pregnancy in cases of patients who wished to conceive at the FU visit, recurrences, reoperation rate, and complications. After surgery, long-term hormonal therapy was offered at the hospital to all women not trying to conceive until a new pregnancy wish to avoid the recurrence of pain or the disease. Patients with a pregnancy wish were counseled for spontaneous trial or direct ART through in vitro fertilization (IVF). We defined endometriosis recurrence as the reappearance or exacerbation of peritoneal disease or lesions in other locations following initial surgical treatment, evaluated through the patient’s history, vaginal examination, and ultrasound.

### 2.5. Statistical Analysis

Data were analyzed using GraphPad Prism 9.0.0. Data analysis included the patients’ ages, surgical procedures, operating times, intraoperative and postoperative complications, and time to discharge. The results were reassumed as the mean and standard deviation for continuous data and as the frequency and percentage for categorical data. The Wilcoxon matched-pairs test for continuous variables assessed the intergroup variations between baseline and FU values. Continuous or quantitative variables were compared using a *t*-test, whereas Fisher’s exact test was applied to compare categorical variables. A *p*-value < 0.05 was considered statistically significant. A forward stepwise multivariate logistical regression analysis was performed to identify potential confounding factors and determine their influence on pain scores, successful pregnancy outcomes, and disease recurrence. Factors analyzed included age, gravidity, previous infertility, previous surgery for endometriosis, and pregnancy after surgery.

## 3. Results

A total of 200 patients who showed symptoms resistant to medical treatment and had received peritoneal endometriosis laser excision within six years at our endometriosis center and with a 24-month FU were included in this study. The average age of the patients was 31 years with an interval of 20–44, and the mean BMI was 22.15 ± 1.4 kg/m^2^. Preoperative clinical characteristics of patients are shown in Table 1.

At the time of surgery, 63% of women were nulliparous, while 37% had at least one pregnancy. Only 19 patients (9.5%) had a previous abdominal surgery for non-related endometriosis causes. Pain-related symptoms were present as an indication for surgery for all our patients, while infertility affected 86 (43%) of women. In the past, all the patients (100%) underwent at least one form of hormonal treatment (combined oral contraceptives or progesterone-only pills). For those continuing therapy without the intention of conception, hormonal treatment was discontinued two months before the surgical procedures. Through preoperative questionnaires regarding symptoms, 83.5% of patients declared to suffer from dysmenorrhea, 56% had chronic pelvic pain, 48.5% reported dyspareunia, 15.5% dyschezia, and 4% dysuria. For the pain level, please see Table 2.

Endometriosis presence was confirmed during surgery in all cases; almost all women showed stage I or II endometriosis (37.5% and 50.5%, respectively) as classified using the rASRM score, while only 12% had stage III. All the patients underwent chromopertubation; bilateral fallopian patency was seen in 71.5% of cases, unilateral patency in 19.5%, and no patency was found in 9%. The surgical procedures performed using a CO_2_ laser were adhesiolysis, biopsy excision, and vaporization. All the patients had histologically confirmed endometriosis, often combined with chronic inflammation and fibrosis. Table 3 shows the numbers and percentages of the surgical findings. The mean operative time was 47 min (range 31–104). No procedures required laparotomy conversion and were completed laparoscopically. The estimated blood loss was 119 ± 51.2 mL. Neither intraoperative nor early or late complications were reported during the whole study. Two patients had a postoperative fever of > 38 °C, which decreased after two days of antibiotic treatment. On average, all the patients were discharged within two days (range 0–3) after surgery. In total, 142 (71%) of the 200 patients were free of analgesic drugs on Day 2.

**Figure 1:**
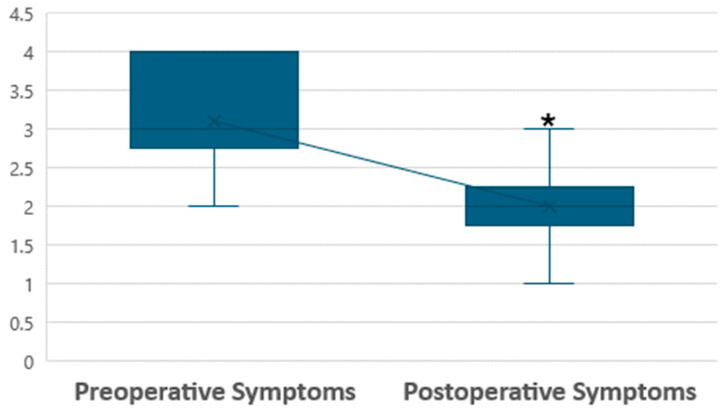
Box plots presented significant differences between symptoms before and after surgery (* *p* < 0.01 vs. preoperative symptoms). Pain score divided into five categories: 5: severe (VAS 8–10), 4: modest (VAS 6–7), 3: moderate (4, 5), 2: mild (3–4), 1: no pain.

A statistically significant improvement in cumulative pain scores was observed at 3, 6, and 12 months of FU (*p* < 0.01) (Figure 1 shows preoperative and postoperative symptoms difference).

**Figure 2:**
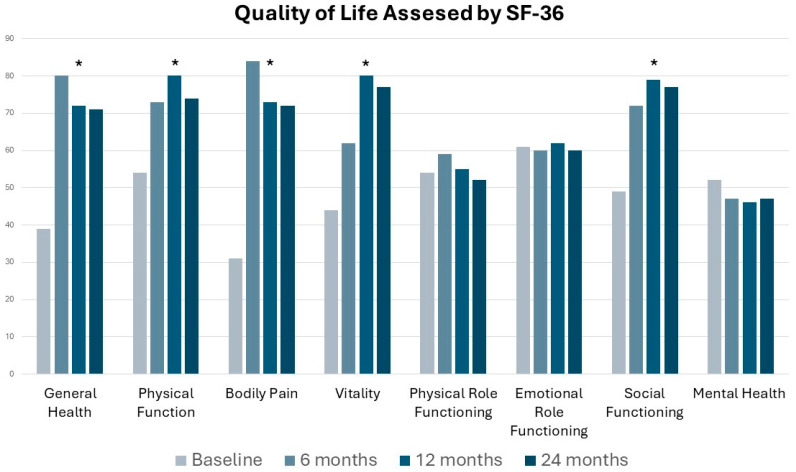
Differences in the patient’s quality of life, as assessed using SF36, before surgery, at 6-, 12-, and 24 months follow-up (* *p* < 0.01 vs. baseline).

Moreover, at a 2-year FU, patients treated showed significant improvement (*p* < 0.01) in five domains of the SF-36 questionnaire, i.e., physical function, general health, pain, vitality, and social functioning (Figure 2). 

**Figure 3:**
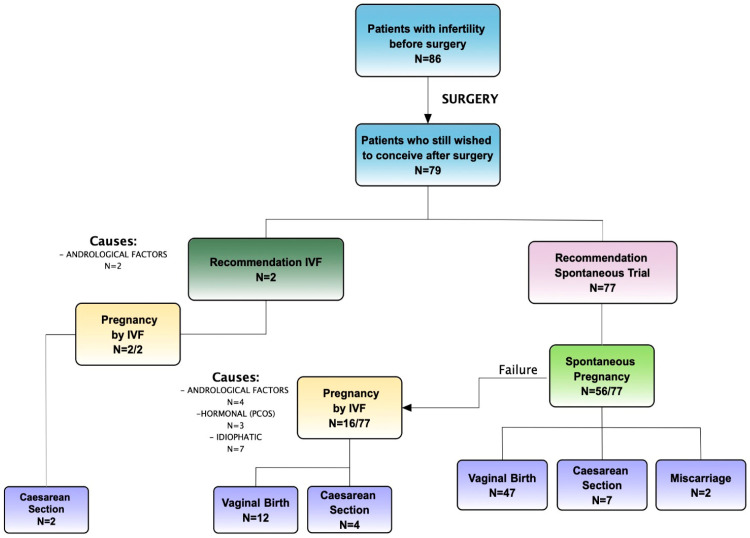
Pregnancy data in infertile patients wishing to conceive with the recommendation, type of pregnancy, and delivery. IVF: in vitro fertilization; PCOS: polycystic ovarian syndrome.

**Figure 4:**
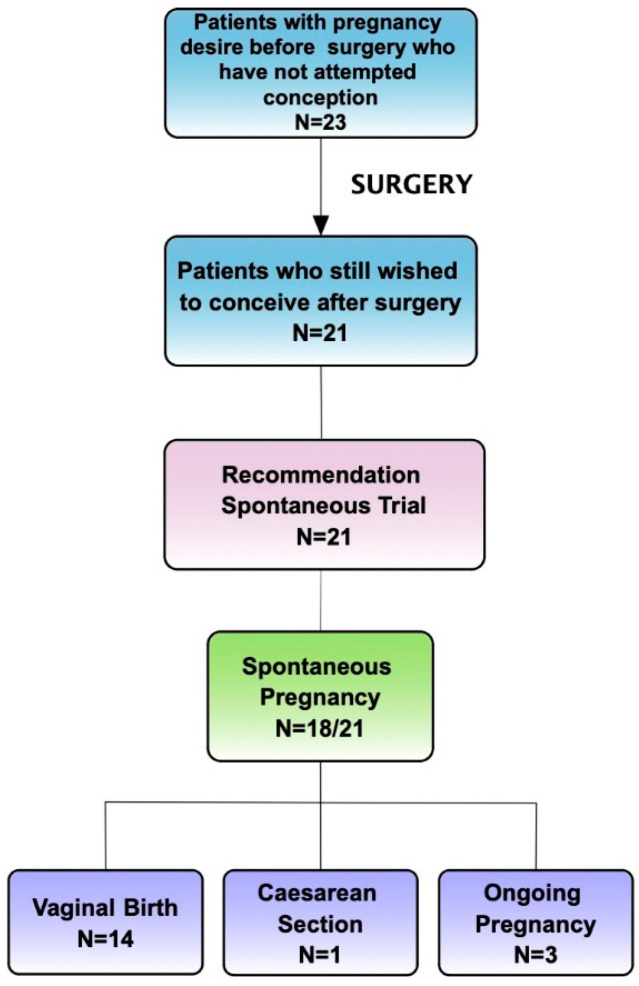
Pregnancy data in patients wishing to conceive who have not attempted conception before surgery with the recommendation, type of pregnancy, and delivery.

At discharge, 110 patients began continuous dienogest administration (2 mg daily) to prevent clinical and symptom recurrences. During FU, some patients interrupted hormonal treatment due to the will to get pregnant. Among patients who did not undergo hormonal therapy right after surgery, 86 (43%) were infertile before surgery, and 79 of them still wished to conceive and tried immediately. A total of 101 patients had a desire for pregnancy after surgery. The overall pregnancy rate among those wishing to conceive was 92/101 (91.1%): 80.4% spontaneous and 19.6% ART, requiring in 2/18 (11.1%) cases immediate IVF because of severe male factor infertility (indication and overall pregnancy outcome detailed in Figure 3 and Figure 4). Overall, the live birth rate was 87/92 (94.5%). In total, 86 patients presented infertility before surgery, and only 79 still wished to conceive after the surgical procedure. Among infertile patients with an active desire for pregnancy, the surgical treatment showed a significant improvement in terms of fertility, with an overall pregnancy rate after surgery of 93.7% (*p*-value < 0.01) with 75.7% natural pregnancies and 24.3% IVF (overall fertility and pregnancy outcome is detailed shown in Table 4).

In the multivariate linear regression analysis among patients with a pregnancy wish, none of the analyzed factors showed a significant effect on pregnancy outcomes in the total population and the infertile group. No significant differences in patient characteristics and surgical data were found in the infertile group among patients who conceived compared with those who failed to conceive. However, the sample size is limited and underpowered to detect eventual differences. The time-to-pregnancy distribution shows that pregnancies (spontaneous or IVF) occur in the first year after surgery. During our FU analysis, we observed five cases of endometriosis recurrence among the study cohort; four of these cases did not initiate hormonal therapy post-surgery despite our insistent recommendation, while the fifth case opted not to begin hormonal treatment initially due to a desire to conceive but subsequent attempts at pregnancy were unsuccessful, and the disease recurred.

## 4. Discussion

The validation of laser usage during laparoscopic procedures, spearheaded notably by Camran Nezhat in the late 1980s, has been significant. Lasers, as instruments emitting coherent light through an optical amplification system based on stimulated electromagnetic radiation discharge, concentrate high power into a minimal area, offering extreme precision in the vaporization of tissue without bleeding, a controlled penetration depth, potent hemostatic properties, the absence of electrical interference, a high safety profile, and favorable patient tolerance. Laser technology is experiencing significant growth and adoption across various fields of minimally invasive gynecological surgery, ranging from hysteroscopy for the treatment of polyps, adhesions, and fibroids [37] to laparoscopy both for superficial and DIE [38]. Surgical intervention remains a cornerstone in the management of endometriosis-associated pain and infertility, particularly for patients unresponsive to hormonal therapy. However, the optimal timing and approach to surgery remain subjects of debate, especially regarding DIE in infertility management vs. medically assisted reproduction treatments [12]. Pain intensity appears independent of disease stage or extent, complicating treatment decision-making. Despite advancements in surgical techniques, such as laser laparoscopy technologies, persistent or recurrent pain following surgical intervention remains a significant concern, necessitating further research into treatment outcomes and long-term efficacy to determine the effectiveness of surgical intervention for SPE.

### 4.1. Primary Outcome: Pain Management

In all cases, endometriosis was macroscopically visible and confirmed during surgery, with most patients presenting with stage I or II disease according to the rASRM score, probably because of the specific selection of our patients with no involvement besides SPE. Surgical procedures using a CO_2_ laser were performed successfully, including adhesiolysis, biopsy excision, and vaporization. Histological analysis confirmed endometriosis in all patients, often accompanied by chronic inflammation and fibrosis. This intervention effectively reduced pain, thereby enhancing the quality of life for patients and improving the chances of pregnancy in women with early stage endometriosis for over one-year post-FU. Dysmenorrhea was the most prevalent symptom in our population and usually benefits from continuous hormonal therapy. Chronic pelvic pain may also persist during hormonal therapy and is associated with a high level of VAS score from the patients. Differently, the few patients suffering from dyschezia or dysuria did not manifest high VAS levels, probably because of the strict selection of our cohort without involving other endometriosis compartments besides the superficial peritoneum. Every surgical procedure was completed laparoscopically without the need for laparotomy conversion. The mean operative time was 47 min, and the estimated blood loss was minimal. This short surgical time length is justified by our operative laparoscopy in conscious sedation technique, which requires experienced surgeons, considering the low pneumoperitoneum pressure and few Trendelemburg degrees used. Thanks to this approach, we obtain a shortened hospitalization of patients, resulting in some cases of discharge on the same day of the surgical procedure. No intraoperative or postoperative complications were reported, indicating the safety and efficacy of the surgical approach. Significant improvements in pain scores were observed during FU, as shown in Figure 1, with a *p*-value < 0.01 between preoperative and postoperative symptoms presented by boxplots. However, achieving these goals necessitates careful patient selection for surgery in endometriosis cases, and optimal timing ensures the most relevant benefits [39]. Moreover, we reported significant improvements (*p*-value < 0.01) in five domains of the SF-36 questionnaire: general health, physical function, bodily pain, vitality, and social functioning at two years post-surgery (Figure 2). These findings in our population highlight the advantages of alleviating pain and improving the overall quality of life. Specifically, we report how decreased pain-related symptoms following surgery positively impact patients’ social interactions. The significant improvement in quality of life among patients treated for endometriosis has been observed through various tangible indicators. For example, many patients have reported increased participation in sports activities, suggesting a restoration of physical functionality and greater satisfaction in facing daily physical challenges. Additionally, we have noted an improvement in interpersonal relationships, with patients reporting increased engagement in social activities with friends and family, likely correlated with the reduction in debilitating symptoms associated with endometriosis. Other signs of improvement include a reduction in the use of pain medication, suggesting better pain management and improved sleep quality. Lastly, many patients have reported increased vitality in tackling work and daily activities, indicating an overall enhancement in well-being and general functionality. However, it is noteworthy that while there was a direct enhancement in social aspects, such improvement did not translate into a corresponding gain in mental health, as assessed using the SF-36 questionnaire. Other findings of this study underscore the scanning-aided CO_2_ laser’s precise cutting capability and minimal heat dispersion. When anatomy was distorted for the presence of complex adhesions, we preferred to associate the laser with the hydro-dissection technique, first developed by Camran Nezhat, which involves using a liquid solution, often saline, to separate tissues while gently vaporizing or removing endometriotic lesions. CO_2_ laser and hydro-dissection allow for the precise and gentle vaporization of endometriotic lesions without damaging surrounding tissues. This technique is advantageous when dealing with lesions close to delicate structures such as the ureters or bowel. We prefer laser vaporization to treat superficial endometriosis over excision or coagulation because of the laser’s ability to provide extreme precision in removing lesions without damaging surrounding tissues. Laser vaporization allows for targeting specific endometriotic lesions, vaporizing them in a controlled manner. This reduces the risk of damaging delicate anatomical structures, such as blood vessels and nerves, which may be compromised during excision or coagulation. Additionally, laser vaporization may reduce the risk of postoperative adhesion formation, as it does not involve the removal of excess tissue. This technique can, therefore, offer advantages in terms of post-operative recovery, reducing the risk of complications and improving long-term outcomes for patients with SPE. Consistent with the literature, we found similar results regarding pain reduction after surgery in a study by Ghai et al. [40], where almost 25% of patients treated for SPE were non-responders. Interestingly, in their cohort, women were more likely to be non-responders if treated for early stage endometriosis compared with those with severe endometriosis. This is probably because the influence of preoperative symptoms of women suffering from severe endometriosis is such that surgery may impact more on pain control. Moreover, we highlight the importance of performing SPE surgery after a pause of at least two months of hormonal therapy. There is a modification of endometriotic lesion size during hormone therapy [41,42], especially with dienogest, that may underestimate SPE extent and leave implants untreated. Several authors reported significant pain control after laparoscopic treatment, with improved social aspects and a step backward in terms of the pain threshold perceived by patients [43,44,45,46], which returned to a pre-disease level independent from the stage of the disease [47]. A limit of several studies is not to focus only on SPE; therefore, the comprehension of its role in influencing pain perceptions remains uncertain. In our cases, this disease localization appears to play an important role, considering the significant results we assessed on pain items post-surgery. We needed to perform an excision biopsy before endometriotic lesions vaporization to have the histological confirmation of the disease; in almost all cases along with endometriosis, we found a chronic inflammation of the peritoneum, as reported by the pathologists. In the work of Dückelman et al. [48], certain patients suffered from persistent pelvic pain after the excision of endometriosis, probably because of associated adenomyosis, a leading cause of dysmenorrhea found in three-quarters of women of their cohort during sonography examinations. We excluded the presence of adenomyosis preoperatively through an accurate ultrasound performed by a skilled sonographer in our center during the presurgical assessment. Therefore, the reason why pain remains in some patients is unknown and requires more consideration. Several patients had pregnancy desires after surgery and refused hormonal therapy; this could be one of the possible explanations regarding postoperative persistent pain. Surgery aims to remove all the visible lesions, and hormonal treatment should prevent some residual diseases from recreating a peritoneal environment for the persistence of pain [49,50]. Although surgery for endometriosis can improve pain and fertility, the risk of disease recurrence is high [51]. Among the 200 treated patients with an FU of 2 years, we had very low recurrences (2.5%), probably because we only treated patients with SPE and excluded any other type of endometriotic localization from our cohort preoperatively. Moreover, we performed surgeries without the downregulation that hormonal therapy may cause to the SPE lesions. Taylor et al. [52] found recurrent endometriotic lesions, especially in the margin of earlier resection areas. In our cases, vaporization allowed us to extend the treated peritoneum area safely, controlling depth energy release in a way that otherwise would be too invasive by increasing the excision area. It is essential to recognize that women with endometriosis frequently encounter several concurrent regional pain disorders, which, when untreated, can worsen or contribute to pelvic pain. We strongly believe that pain persistency after surgical treatment could be related to the intrinsic nature of endometriosis as a chronic inflammatory disease leading to an up-regulation of pain sensitization promoting cytokines, nociceptive, and neuropathic pathways activation [53,54]. On the contrary, some authors suggested that endometriosis progression, growth, and invasion are related to an indispensable role of anti-inflammatory cytokines [55]. This reflects the poor comprehension of the real endometriosis etiology and pathogenesis and how a targeted medical or surgical treatment remains today not applicable. The complexity around pain perception probably reflects the heterogeneity of its cause, including mental health as an essential factor to consider. Indeed, endometriosis is often associated with other comorbidities, which could disorientate the clinician to a prompt and adequate treatment [56]. We invite gynecologists to have a multidisciplinary care model approach to patients suffering from endometriosis, promoting psychological therapies, nutrition advice, and cooperation with rheumatologists and gastroenterologists. Recent research has explored the effectiveness of laparoscopic treatment for endometriosis, comparing it with diagnostic laparoscopy or medical approaches. While a Cochrane review examined a limited number of randomized controlled trials [57,58] comparing surgical intervention with diagnostic laparoscopy alone [59], the overall findings were inconclusive regarding the impact of laparoscopic surgery on overall pain levels and quality of life due to the varying quality of these studies. In contrast with our findings, another recent systematic review and meta-analysis by Arcoverde et al. [60] indicated that surgery for endometriosis significantly improved mental component scores (MCS) but not physical component scores (PCS). Similarly, Vercellini et al. [61] found notable enhancements in health-related quality of life (QoL) and sexual satisfaction scores following surgery. Still, they did not reduce the medium- or long-term frequency and severity of the recurrence of dysmenorrhea. Many authors have used different types of lasers in different endometriosis compartments, achieving promising results yet to be confirmed [62], and even if no clinical trials have specifically investigated the impact of surgical intervention on pain symptoms in cases of SPE, the European Society of Human Reproductive Medicine (ESHRE) Guidelines for Endometriosis recommends offering surgery as one of the options to reduce endometriosis-associated pain. The ongoing ESPriT2 trial in the UK further advances SPE research by focusing on women diagnosed solely with SPE during diagnostic laparoscopy. Random assignment compares outcomes between surgical removal of SPE and diagnostic laparoscopy alone, aiming to determine the efficacy and safety of surgical intervention for this subset of patients. Further results will determine the correct management and impact of surgery on reducing pain in patients with SPE.

### 4.2. Secondary Outcome: Overall Pregnancy Rate Assessment

Among patients desiring pregnancy after surgery, a high pregnancy rate of 91.1% was achieved, with most pregnancies occurring spontaneously (80.4%). The overall live birth rate was 94.5%, indicating successful fertility outcomes following surgical treatment for endometriosis. Our results demonstrated a significant improvement in fertility outcomes among infertile patients with an active wish to conceive following surgical treatment, with an overall pregnancy rate of 93.7% (*p*-value > 0.01) with 75.7% natural pregnancies and 24.3% IVF (please see Table 4). Continuous administration of dienogest post-surgery was associated with a reduced risk of clinical and symptom recurrences, although some patients interrupted hormonal treatment to pursue pregnancy desire. The decision to use hormonal therapy post-surgery should be individualized based on the patient’s reproductive goals. Figure 3 and Figure 4 show pregnancy data in patients wishing to conceive with the recommendation, type of pregnancy, and delivery in detail. During the FU period, inquiries were made regarding any new developments or factors related to male infertility, but no new information emerged from the partners besides the initial ones. Direct IVF was indicated for severe male factors, while IVF after the failure of spontaneous attempts was for women with hormonal or idiopathic causes. In total, 9% of women had no tubal patency bilaterally; this could explain some cases of female fertility impairment and the need for IVF. Multivariate analysis did not identify significant factors influencing pregnancy outcomes among patients desiring pregnancy. According to the literature, the timing of pregnancy post-surgery in our cohort suggests that most pregnancies occur within the first year following surgery. Tahmasbi Rad et al. [63] concluded that the first 12 months were the optimal time for pregnancy. For women with rASRM stages I and II, spontaneous pregnancy can probably be delayed for up to 24 months, but in patients with rASRM stages III and IV, ART may be considered after 12 months. Although very encouraging, these results, especially in the infertile population, do not permit us to conclude the benefit of surgery compared with direct IVF, given the lack of a control group. However, they support the idea that SPE vaporization does not affect future fertility. In a recent study by Nezhat C. et al. [64], they investigated the prevalence of endometriosis in patients with unexplained infertility with a result of over 90%, as defined as the presence of endometrial-like glands and stroma, in their pathology reports. The high prevalence demonstrated raises the natural question: “Are we potentially under-detecting endometriosis? Furthermore, is there a direct link between endometriosis and infertility?”. This finding highlights the critical importance of early and accurate diagnosis and the necessity for personalized treatment approaches.

**Figure 5:**
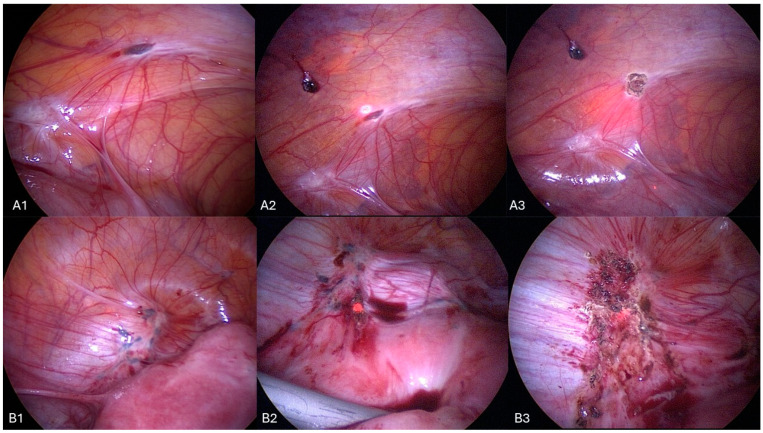
CO_2_ laser vaporization, (**A1**,**A2**,**A3**) and (**B1**,**B2**,**B3**) sequences show different lesions and the procedure for their removal from the beginning to the final result.

Overall, our study underscores the safe and valid approach of CO_2_ laser vaporization (see Figure 5) and the importance of surgical intervention in managing endometriosis, with favorable outcomes observed in pain relief, quality of life improvement, and fertility restoration. Further research with larger sample sizes is needed to validate these findings and identify potential predictors of surgical success and pregnancy outcomes in patients with endometriosis.

## 5. Conclusions

Our cohort focused on a large number of SPE patients, excluding every type of different localization. We had histological confirmation for every patient included in our analysis. Our study utilized a validated questionnaire to uniformly document clinical data and pain history, ensuring consistency and reliability in data collection. All surgical interventions were performed using two high-volume minimally invasive gynecologic surgeons with extensive experience with CO_2_ laser, using standardized procedures to ensure uniformity and expertise in surgical technique throughout the study. All patients discontinued standard medical suppression treatment for endometriosis at least two months before surgery, reducing the potential confounding effects of ongoing treatment on surgical outcomes. The study achieved an FU of two years, enhancing the reliability of postoperative assessments like complications and recurrences. The principal limit of our study is the need for a control group to provide the effectiveness of the intervention; the frequent association of pain symptoms and infertility makes it challenging to constitute a correct control group. Indeed, hormonal treatments are not an option for patients wishing to conceive, and it is difficult to propose simple observations in the case of pain symptoms. Retrospective diagnosis of infertility poses a limitation when assessing the overall fertility rate because it relies on patients’ recollection of their fertility status, which may be subject to many biases. We acknowledge the limitation of the study’s sample size, which may be underpowered to detect eventual differences accurately, and caution readers to interpret the findings considering this constraint. This highlights the importance of large-scale, prospective, longitudinal studies to confirm and further elucidate the observed trends.

## Figures and Tables

**Table 1:** Preoperative characteristics of the patients.

Parameter	*N*:200
Age, (yrs), median (interval)	31 (20–44)
Body mass index, kg/m^2^ mean ± SD	22.15 ± 1.4
Nulliparous, *n* (%)	126 (63%)
Multiparous, *n* (%)	74 (37%)
Indication for surgery, *n* (%)	
Pain-related symptoms	200 (100%)
Infertility	86 (43%)
Prior abdominal surgery (appendectomy, ectopic pregnancy, cholecystectomy, and emergency diagnostic laparoscopy), *n* (%)	19 (9.5%)
Prior medical treatment, *n* (%)	200 (100%)

**Table 2:** Presurgical symptoms.

Symptoms, *n* (%)	
Chronic pelvic pain	112 (56%)
Dysmenorrhoea	167 (83.5%)
Dyspareunia	97 (48.5%)
Dyschezia	31 (15.5%)
Dysuria	8 (4%)
Strength of symptoms preop. Average and max. (median)	
Chronic pelvic pain	6 and 8
Dysmenorrhoea	6 and 9
Dyspareunia	4 and 8
Dyschezia	3 and 5
Dysuria	2 and 6

**Table 3:** Intraoperative findings.

Surgical Findings	
Adhesiolysis	69 (34.5%)
Dye Test	200 (100%)
No Patency	18 (9%)
Patency both sides	143 (71.5%)
Patency one side	39 (19.5%)
rASRM I	75 (37.5%)
rASRM II	101 (50.5%)
rASRM III	24 (12%)
Intraoperative complications	0 (0%)
Conversion to laparotomy	0 (0%)
Mean operative time (min), mean (interval)	47 (31–104)
Estimated blood loss, ml mean ± SD	119.2 ± 51.2
Hospital stay (days), mean (interval)	2 (1–3)
Hormonal therapy after surgery (dienogest 2 mg), *n* (%)	110 (55%)
Postoperative pregnancy intent, *n* (%)	101 (50.5%)
Number of patients analgesic-free at day 2, *n* (%)	142 (71%)
Complications according to Clavien–Dindo classification, *n* (%)	None

**Table 4:** Fertility data in both infertile patients wishing to conceive after surgery and the overall population.

Fertility Outcome after Surgery	
The overall pregnancy rate in patients wishing to conceive, *n* (%)	Patients wanting to conceive after surgery; *N* = 101
- Total	92/101 (91.1%)
- Spontaneous	74/92 (80.4%)
- ART	18/92 (19.6%)
Overall pregnancy outcome in patients wishing to conceive, *n* (%)	
- Vaginal Birth	73/92 (79.3%)
- Caesarean Section	14/92 (15.2%)
- Miscarriage	2/92 (2.2%)
- Ongoing pregnancy	3/92 (3.3%)
Overall birth rate in patients wishing to conceive, *n* (%)	87/92 (94.5%)
Overall pregnancy rate in the infertile population after surgery, *n* (%)	Infertile patients wishing to conceive after surgery; *N* = 79
- Total	74/79 (93.7%)
- Spontaneous	56/74 (75.7%)
- ART	18/74 (24.3%)
Overall pregnancy outcome in infertile patients wishing to conceive, *n* (%)	
- Vaginal Birth	59/74 (79.7%)
- Caesarean Section	13/74 (17.6%)
- Miscarriage	2/74 (2.7%)
- Ongoing pregnancy	0/74 (0%)
Overall birth rate in infertile patients wishing to conceive, *n* (%)	72/74 (97.3%)
Mean time to pregnancy (weeks), mean ± SD	11.3 ± 3.2

## Data Availability

The data cannot be shared publicly due to privacy restrictions imposed by the author’s affiliated institution.
